# Computer-Assisted System in Stress Radiography for Anterior Cruciate Ligament Injury with Correspondent Evaluation of Relevant Diagnostic Factors

**DOI:** 10.3390/diagnostics11030419

**Published:** 2021-03-02

**Authors:** Chien-Kuo Wang, Liang-Ching Lin, Yung-Nien Sun, Cheng-Shih Lai, Chia-Hui Chen, Cheng-Yi Kao

**Affiliations:** 1 Department of Medical Imaging, National Cheng Kung University Hospital, College of Medicine, National Cheng Kung University, No. 1 University Rd, Tainan 704, Taiwan; n625396@mail.hosp.ncku.edu.tw (C.-K.W.); laics@mail.ncku.edu.tw (C.-S.L.); 2 Department of Statistics, National Cheng Kung University, No. 1 University Rd, Tainan 704, Taiwan; lclin@mail.ncku.edu.tw; 3 Department of Computer Science and Information Engineering, National Cheng Kung University, No. 1 University Rd, Tainan 704, Taiwan; ynsun@mail.ncku.edu.tw; 4 Department of Medical Imaging and Radiological Sciences, I-Shou University, No.8, Yida Rd., Jiaosu Village, Yanchao District, Kaohsiung City 82445, Taiwan

**Keywords:** knee, anterior cruciate ligament, ACL, stress radiography, computer-aided diagnosis

## Abstract

We sought to design a computer-assisted system measuring the anterior tibial translation in stress radiography, evaluate its diagnostic performance for an anterior cruciate ligament (ACL) tear, and assess factors affecting the diagnostic accuracy. Retrospective research for patients with both knee stress radiography and magnetic resonance imaging (MRI) at our institution was performed. A complete ACL rupture was confirmed on an MRI. The anterior tibial translations with four different methods were measured in 249 patients by the designed algorithm. The diagnostic accuracy of each method in patients with all successful measurements was evaluated. Univariate logistic regression analysis for factors affecting diagnostic accuracy of method four was performed. In the inclusive 249 patients, 177 patients (129 with completely torn ACLs) were available for analysis. Mean anterior tibial translations were significantly increased in the patients with a completely torn ACL by all four methods, with diagnostic accuracies ranging from 66.7% to 75.1%. The diagnostic accuracy of method four was negatively associated with the time interval between stress radiography and MRI as well as force-joint distance on stress view, and not significantly associated with age, gender, flexion angle, intercondylar distance, and force-joint angle. A computer-assisted system measuring the anterior tibial translation in stress radiography showed acceptable diagnostic performance of complete ACL injury. A shorter time interval between stress radiography and MRI as well as shorter force-joint distance were associated with higher diagnostic accuracy.

## 1. Introduction

The anterior cruciate ligament (ACL) is the main restraint of anterior tibial translation. It may be torn when taking unexpected heavy loading, e.g., strenuous exercise or traffic accident, thereby causing knee instability [1]. Therefore, early diagnosis of ACL tear is crucial. Magnetic resonance imaging (MRI) with high resolution and good imaging quality is helpful for confirming the diagnosis of the ACL tear, but it is expensive and time-consuming [2]. Clinical diagnosis of ACL tear is still important.

The clinical diagnosis of ACL tear is based on anterior tibial translation by applying a different force to the knee. The anterior drawer test is subjective, unmeasurable, and not well reproducible [3]. Stress radiography of the knee offers a relatively objective and measurable approach to quantify the anterior tibial translation when applying force to the knee [4,5,6,7]. A prior study has focused on the effect of knee position on the reproducibility of measurements taken from stress radiography [8]. A different knee flexion angle, injury extent, muscle guarding, and femoral rotation affect the laxity measurement using stress radiography [4,9,10]. Another study compared different measuring methods and assessed the intra-class correlation coefficient of each method to evaluate the knee instability [11]. However, the reliability of manual measurements done by different readers and different methods is questionable.

Several automatic methods for objective measurement of joint space or angle using computer-assisted systems have recently been developed [12,13,14]. Automatic computer image assessment and analysis system can segment the femur, tibia, and patella using an edge-based method. Then, some landmarks to calculate tibial translation distance can be defined using a segmentation algorithm.

In this study, we designed a computer-assisted system that automatically segmented bone contours of the knee and, subsequently, measured the distance of anterior tibial translation in stress radiography. The diagnostic performances of different methods for ACL injury were evaluated. It was hypothesized that we could use our computer-assisted system to (1) evaluate the diagnostic performance of stress radiography for ACL insufficiency by different measurement methods, and (2) assess the effects of age, gender, time interval between stress radiography and MRI, and technical factors on the diagnostic accuracy.

## 2. Materials and Methods

### 2.1. Patients

**Figure 1:**
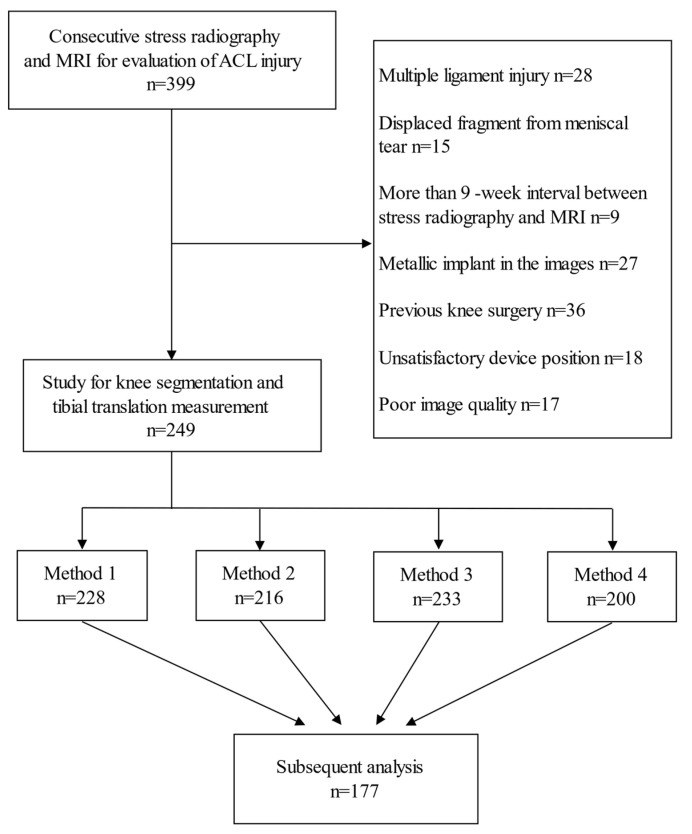
The flow chart of patient inclusion and exclusion.

Institutional Review Board approval was obtained, and the requirement to obtain informed consent was waived. A retrospective search of the Picture Archiving and Communication System (PACS) for patients with both knee stress radiography by Telos device (Metax, Hungen, Germany) and MRI at our institution was performed. The MRI was reviewed by one musculoskeletal radiologist, and the status of ACL was classified into complete tear, partial tear, or no tear. If the review did not match the previous report, a musculoskeletal radiologist with more than 20 years of experience made the final decision. The patients were divided into one group with a complete ACL tear, and another group without a complete ACL tear. The time interval between stress radiography and MRI was recorded. A total of 399 patients with stress radiography and MRI for evaluation of ACL injury were included in this study, from which 150 patients were excluded (Figure 1). In addition, 28 patients were diagnosed as multiple ligamentous injuries on MRI, while 15 patients with the torn meniscus had displaced fragments. Nine patients were excluded due to long interval (>9 weeks) between stress radiography and MRI. Furthermore, 27 patients were excluded due to anatomic shape changes by severe osteoarthritis, fractures, or intraarticular loose bodies, 36 patients were excluded due to a previous knee surgery, 18 patients were excluded because of unsatisfactory device position to obscure the segmentation, and another 17 patients were excluded due to poor image quality. Imaging data processing and a tibial translation measurement were performed in the remaining 249 patients.

### 2.2. Knee Segmentation

**Figure 2:**
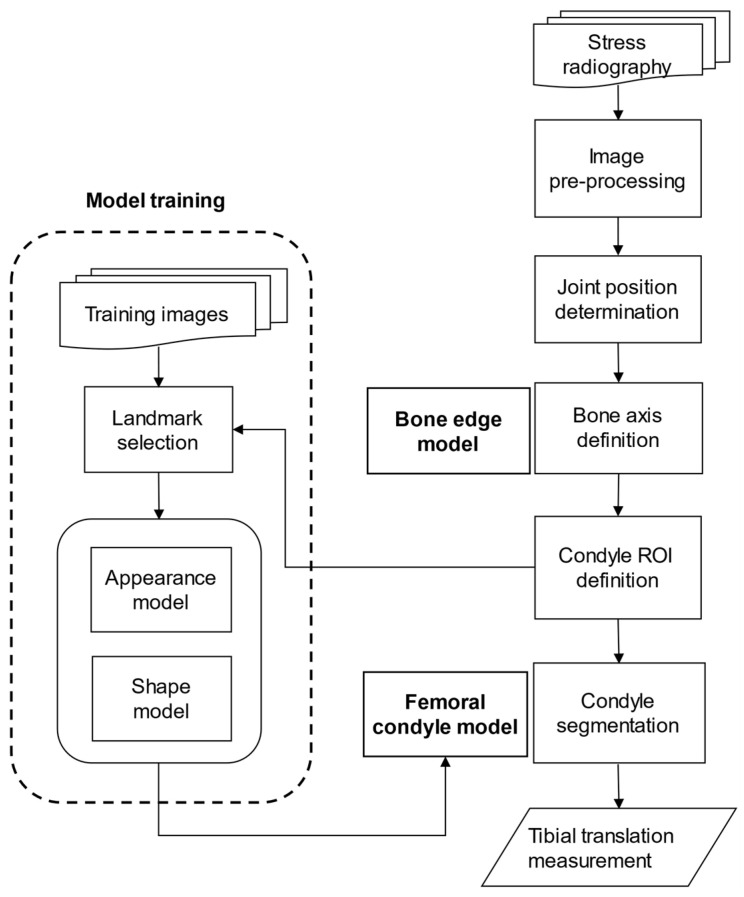
The flow chart of knee segmentation.

**Figure 3:**
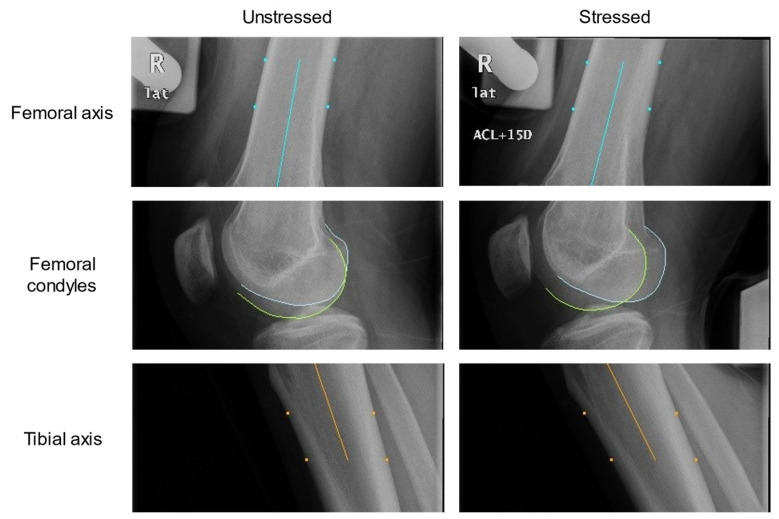
The segmentation results of stress radiography for anterior cruciate ligament (ACL) injury. In this case, the lateral (lat) projection views of the right (R) knee before and after exerting a force of 15 daN (+15D) posteriorly on the tibia by a stress device were taken. The anterior edge and the posterior edge of both femur and tibia were segmented (cyan and orange dots) and used to calculate the femoral axis and the tibial axis (cyan and orange straight lines). The condyle contours (white and yellow curves) were also segmented.

Lateral projection views of the knee before and after exerting a force of 15 daN (1 daN = 10 N), in accordance with a previous study [15], posteriorly on the tibia by a stress device were taken. In the automatic measurement of knee translation in stress radiography, the most important and challenging part was knee segmentation (Figure 2). We first performed down sampling of the images to improve the efficiency of following pre-processing. Then, the Gaussian smoothing is applied to the images for noise removal, making the edge of the bone relatively clear by removing the artifact in the image. Since the knee joint is located between two major bony structures (femur and tibia), a less bony structure meant less brightness. For every row in the image, the sums of all gray values were computed. The row with a local minimum was determined as the knee joint location and taken as a reference position. To segment the contours in the complex X-ray image, we used the framework of minimal intensity and a shape cost path model proposed by D. Seghers et al. [16]. The anterior edge and the posterior edge of both femur and tibia were segmented and used to calculate the femoral axis and the tibial axis by the bone edge model. The femoral axis was drawn as the midline of the anterior and posterior edges of the femur between an 8–14-cm distance away from the joint space, while the tibial axis was drawn similarly. Afterward, the joint position and femoral axis were used to define the region of interest (ROI) for femoral condyles. A condyle, which is most fitted to the appearance and shape in the image, is segmented. The femoral condyle model was built from a set of training images based on the framework of the appearance model and shape model proposed by D. Seghers et al. [16]. The condyle contour was segmented within the ROI by the femoral condyle model. Finally, we could define the landmark points on the bone axes and condyle contours (Figure 3).

### 2.3. Methods to Measure Tibial Translation

Four methods measuring the anterior tibial translation were adopted in this study. The first two methods were introduced by Wirz et al. [8], and the latter two methods were proposed by this study. The peripheral landmarks are shown as inaccurate if the rotation is changed, and central methods are the most affected if flexion is altered [17]. In all four methods, the femoral reference location and the tibial position were defined. Finally, the tibial translation was determined as the change in the tibial position.

**Figure 4:**
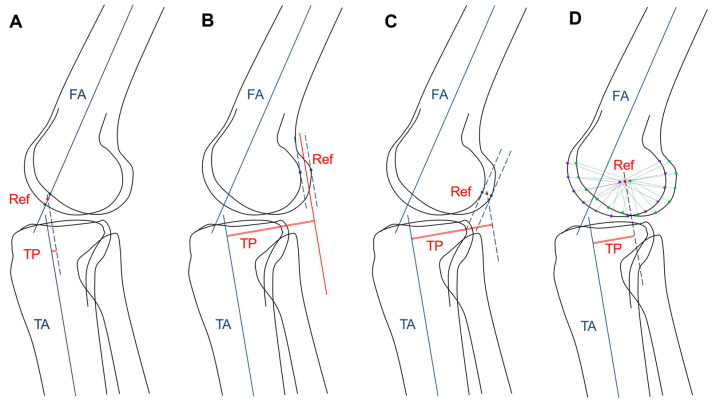
Four measurement methods. The femoral axis (FA) and the tibial axis (TA) were drawn first. (**A**) In method 1, the midpoint of the intersection points of the femoral axis and the femoral condylar edges was the femoral reference point (Ref). (**B**) In method 2, the midline of the lines tangential to posterior femoral condylar edges and parallel to TA was the femoral reference line (Ref). (**C**) In method 3, the midpoint of the tangent points of the lines tangential to posterior femoral condylar edges and parallel to FA was the femoral reference point (Ref). (**D**) In method 4, the midpoint of the center points of the medial and lateral femoral condyle landmarks was the femoral reference point (Ref). Finally, in all four methods, the distance between the TA and Reference was the tibial position (TP).

The central measurement (method 1) is illustrated in Figure 4A. The points where the femoral axis intersected the margins of the medial and lateral femoral condyles were found, and the femoral reference point was defined as the midpoint of the two intersection points. The tibial position was defined as the distance between the femoral reference point and the tibial axis.

A combined central-peripheral measurement (method 2) is illustrated in Figure 4B. The lines parallel to the tibial axis and tangential to the posterior margins of the medial and lateral femoral condyles were drawn, and the femoral reference line was defined as the midline of the two tangent lines. The tibial position was defined as the distance between the femoral reference line and the tibial axis.

Femoral condyle tangent measurement (method 3) was modified from method 2 and illustrated in Figure 4C. Instead of using a line parallel to the tibial axis, the lines parallel to the femoral axis and tangential to the posterior margins of the medial and lateral femoral condyles were drawn, and the femoral reference point was defined as the midpoint of the two tangent points. The tibial position was defined as the distance of the femoral reference point and the tibial axis.

The condyle center measurement (method 4) was newly proposed and is illustrated in Figure 4D. The landmarks that represented the medial and lateral femoral condyle margins were first defined by a femoral condyle model. The center points of the medial and lateral femoral condyle landmarks were obtained, and the femoral reference point was defined as the midpoint of the two center points. The tibial position was defined as the distance of the femoral reference point and the tibial axis.

In all four methods, the segmentation results were reviewed by one musculoskeletal radiologist to search for inappropriate landmarks (femoral axis, tibial axis, and the margins of femoral condyles) obtained by the segmentation algorithms. The segmentation result of each measurement method was only considered successful if all landmarks were appropriate.

### 2.4. Measurements of Technical Factors

Stress radiography of the knee could be affected by various technical factors. Changes in flexion and rotation occur as the knee joint is stressed. To investigate the technical factors affecting the result of tibial translation in stress radiography, we additionally measured the knee flexion angle, intercondylar distance, force-joint distance, and force-joint angle.

The knee flexion angle was defined as the angle between the femoral axis and the tibial axis. The intercondylar distance was defined as the distance between the center points of the medial and lateral femoral condyle landmarks segmented by the femoral condyle model. The force-joint distance was defined as the distance between the tibial plateau and the skin point at the posterior load central line, and reported as positive and negative values for the skin point below and above tibial plateau respectively. The force-joint angle was defined as the angle between the tibial plateau and the posterior load central line, and reported as positive and negative values for the direction of force toward and away from the tibial plateau, respectively.

### 2.5. Statistical Analysis

Analyses were conducted using statistical software (R for Windows, version 3.5.2). The receiver operating characteristic (ROC) curves were performed to illustrate the diagnostic abilities of these four methods. The optimal thresholds and cutoff points were obtained by finding the maximum Youden’s *J* statistic, which was defined as the sums of sensitivity and specificity subtracting 1. Youden’s *J* statistic ranged from zero to one with larger values representing better performance. From these cutoff points, the sensitivity, specificity, positive predictive value, negative predictive value, and accuracy were calculated for all four methods.

Sample size calculation was based on the area under the ROC curve (AUC) of 0.8 for stress radiography with the Telos device used in a previous study [9]. An *α* error of 5% and a *ß* error of 20% were accepted to detect any significant difference. On the basis of these calculations, the required sample size was 28 per group, with a 0.5 null hypothesis value and a 1:1 allocation.

To evaluate the effect of different factors on the accuracy, we chose method four, a central measuring method, to minimize the effect of knee rotation during stress. We built a logistic regression model. The odds ratios and the corresponding 95% confidence intervals were evaluated to quantify the strength of the association between each factor and the judgement of method four.

Continuous variables were reported as means ± standard deviations and analyzed with the *t*-test, whereas categoric variables were analyzed with the chi-square or Fisher’s exact test. A *p* value ≤ 0.05 was considered significant.

## 3. Results

This section may be divided by subheadings. It should provide a concise and precise description of the experimental results, their interpretation, and the experimental conclusions that can be drawn. In the inclusive 249 patients, the successful numbers of knee segmentation and tibial translation measurement were 228, 216, 233, and 200 patients by methods one, two, three, and four, respectively, with successful rates ranging from 80.3% to 91.6%. Examples of unsuccessful cases are shown in Appendix A. The final data available for analysis were derived from the 177 patients (mean age ± standard deviation, 28.4 years ± 10.5, age range, 16–68 years) with successful measurements by all four methods. The patient characteristics of our study were summarized in Table 1. Among the 177 patients (144 males and 33 females), completely torn ACLs were found in 129 patients. The mean interval between stress radiography and MRI was 20.44 days.

The mean tibial translation measurements were 10.50 mm ± 5.27, 10.58 mm ± 4.83, 8.85 mm ± 5.02, and 9.78 mm ± 4.89 for patients with completely-torn ACL by all four methods sequentially and respectively. However, those were 6.49 mm ± 3.46, 6.77 mm ± 3.17, 5.07 mm ± 2.80, and 5.82 mm ± 2.93 for patients without completely torn ACL by the four methods respectively. The differences between patients with and without complete ACL injury were all significant for the four methods (*p* < 0.001).

**Figure 5:**
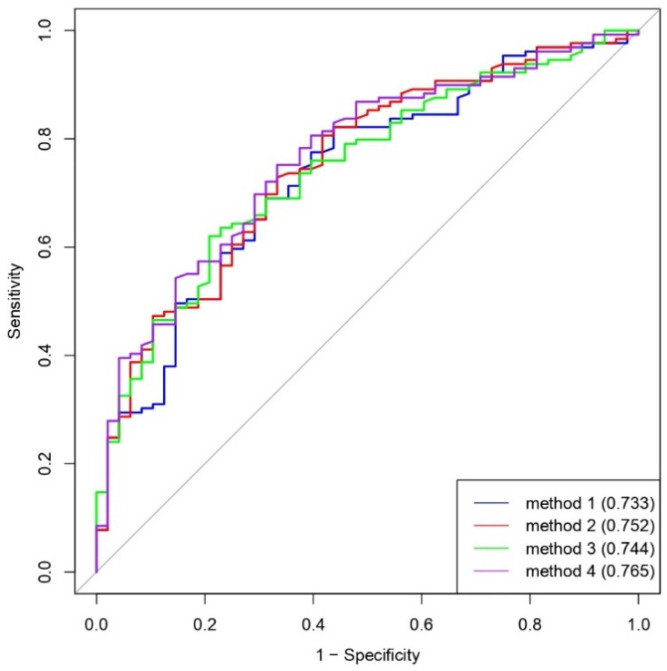
The receiver operating characteristic (ROC) curves of all four measurement methods.

The ROC curves showing the diagnostic abilities of all four measurement methods were illustrated in Figure 5. The AUCs of methods 1 to 4 were 0.733, 0.752, 0.744, and 0.765, respectively. The optimal thresholds and cutoff points (maximum Youden’s *J* statistic) of methods 1 to 4 were 6.16 mm, 7.47 mm, 6.41 mm, and 3.97 mm, respectively. Based on these optimal thresholds, the diagnostic performances of all four measurement methods were shown in Table 2. The accuracies of the four methods were 75.1%, 71.2%, 66.7%, and 72.9%, respectively.

A summary of the results of the univariate analyses for factors affecting diagnostic accuracy of method 4 was presented in Table 3. Among these factors, the time interval between stress radiography and MRI (*p* = 0.035), and force-joint distance on stress view (*p* = 0.032) were negatively associated with the diagnostic accuracy of method 4. A decrease of 1 day in the interval between stress radiography and MRI could increase the diagnostic accuracy by 2.5%. For every 1-mm decrease in the force-joint distance on stress view, 3.3% improvement in diagnostic accuracy was shown. The factors including age, gender, flexion angle, intercondylar distance, and force-joint angle were not significantly related to the diagnostic accuracy using method 4 (*p* = 0.380, *p* = 0.681, *p* = 0.201, *p* = 0.971, and *p* = 0.637, respectively).

## 4. Discussion

The aim of the current study was to measure the anterior tibial translation of knee stress radiography using an automatic computer-assisted system. According to our retrospective study, the anterior tibial translation in the completely torn ACL patients was significantly increased for all four different methods. Furthermore, we found that a shorter time interval between stress radiography and MRI, and closer force-joint distance were associated with higher diagnostic accuracy using a Condyle center measurement (method 4). 

Previous studies have shown that the ACL can regain continuity after partial or complete rupture [18,19,20]. In total, 42% of complete ruptures have been reported to regain continuity after three months [20] and 68% of partial ruptures can regain continuity after 6–36 months [19]. Progression and improvement of ACL tear between the two imaging examinations could contribute to the false negative and false positive cases. Therefore, the negative association between the time interval and diagnostic accuracy was depicted in our study. The effect of the force-joint distance could be explained by the fact that the force exerted on the knee joint in stress radiography was inevitably resorbed by the underlying muscles. A longer force-joint distance resulted in a softer tissue of the lower leg being compressed. Then less net force was exerted to the knee joint, which, in turn, lowered the diagnostic accuracy in our study.

Computer-aided diagnosis (CAD) systems have been used to measure a tibio-femoral angle, joint space narrowing, and osteophyte formation for quantification of knee osteoarthritis severity in previous studies [12,13,14]. The reliability by any manual operation is questionable. The intra-rater and interrater intraclass correlation coefficients should be evaluated in manual measurements. The knee segmentation and landmark point definition in our study worked successfully in more than 80% of the cases for the four methods and are, thus, applied in clinical settings for feasibility in the future.

Four different measurement methods for anterior instability in the stress radiography of knee joint were compared in our study. Wirz et al. have found that the central measuring method and central peripheral measuring method are less affected by internal and external rotation of the knee than the peripheral measuring method [8]. In our study, the methods could be categorized as a central measuring method (methods 1 and 4) and a central-peripheral measuring method (methods 2 and 3). The lower successful rate of method 4 than those of the other three methods might result from the complexity of the segmentation algorithm in the medial and lateral femoral condyle margins. However, the choices of different landmarks and methods in our study did not significantly influence the diagnostic accuracies and AUCs of the four methods.

Lee HJ et al. have investigated how different knee flexion positions affect the diagnostic value of stress radiography for anterior instability and found that the AUCs of stress radiography at 30°, 45°, 60°, and 90° positions are 0.897, 0.78, 0.665, and 0.432, respectively [9]. The tension of ACL increases as the knee flexion angle decreases from 45° to 0° with the greatest tension in the hyperextension. The negative correlation between the flexion angle and the diagnostic accuracy was shown in our study, but could not reach statistical significance. This could be explained by the fact that 71.8% of our cases had a flexion angle less than 45°, and the mean knee flexion angle was 38.47°. A negative correlation might be better shown if our data included more cases with flexion angles of more than 45°.

In this study, we used the distance of anterior tibial translation of the diseased knee to evaluate its anterior instability, instead of a side-to-side difference as in other previous studies [9,21,22,23]. In our institution, MRI is performed only on diseased knees in most cases. Therefore, the condition of ACLs of the contralateral knees could not be presumed to be normal.

In a previous cadaveric study, medial meniscus posterior horn longitudinal tears ranging from 2.4 to 3.3 cm in ACL-deficient knees have resulted in a significant increase in anterior-posterior tibial translation at all flexion angles except 90° [24]. In our study, a non-displaced meniscus tear would not be the reason of the false negative case in detecting the ACL tear. However, the displaced fragments from a torn meniscus could cause locking of the knee joint during stress and, subsequently, reduce the tibial translation. Therefore, patients with displaced fragments of torn meniscus were selectively excluded in our study.

Several issues result in limitations of this study. First of all, the study was retrospective and, therefore, prone to selection bias. Secondly, our algorithm could not clearly identify bone contours with metallic implants in the images, and, thus, had limited utility in postoperative cases. Thirdly, the timing of injury of the study population was unavailable, so further analysis about acute and chronic injury could not be performed. Finally, the reference standard of ACL tear in this study was based on MRI. The partial tear was not separately categorized because of insufficiency to differentiate a partial tear from mucoid degeneration of ACL only by MRI [15]. Moreover, the discrepancy between MRI and arthroscopy could lead to misclassification.

## 5. Conclusions

We have successfully designed a computer-assisted system, which automatically measured the distance of tibial translation in stress radiography with acceptable diagnostic performance. Further analysis showed a shorter time interval between stress radiography and MRI, and shorter force-joint distance were associated with higher diagnostic accuracy.

## Figures and Tables

**Table 1:** Clinical data in 177 patients with knee stress radiographies.

Parameter	Value
Mean age (y)	28.4 ± 10.5 (16–68)
Male gender *	144 (81.4)
Interval between stress view and MRI (d)	20.44 ± 13.80 (0–61)
Flexion angle (°) on stress view	38.47 ± 11.22 (11.88–63.86)
Intercondylar distance (mm) on stress view	4.53 ± 3.61 (0–16.73)
Force-joint distance (mm) on stress view	39.70 ± 11.74 (11.19–67.22)
Force-joint angle (°) on stress view	−10.58 ± 9.30 (−32.14–12.51)
Torn ACL on MRI *	129 (72.9)

Note—Unless otherwise indicated, data are reported as means and standard deviations, with ranges in parentheses. * Data are numbers of cases, with percentages in parentheses.

**Table 2:** Diagnostic performance of knee stress radiography for the detection of anterior cruciate ligament tear.

	Sensitivity	Specificity	PPV	NPV	Accuracy
	percentage (95% confidence interval)				
Method 1	106/129, 82.2 (76.5–87.8)	27/48, 56.3 (48.9–63.6)	106/127, 83.5 (78.0–88.9)	27/50, 54.0 (46.7–61.3)	133/177, 75.1 (68.8–81.5)
Method 2	94/129, 72.9 (66.3–79.4)	32/48, 66.7 (59.7–73.6)	94/110, 85.5 (80.3–90.6)	32/67, 47.8 (40.4–55.1)	126/177, 71.2 (64.5–77.9)
Method 3	80/129, 62.0 (54.9–69.2)	38/48, 79.2 (73.2–85.1)	80/90, 88.9 (84.3–93.5)	38/87, 43.7 (36.4–51.0)	118/177, 66.7 (59.7–73.6)
Method 4	97/129, 75.2 (68.8–81.6)	32/48, 66.7 (59.7–73.6)	97/113, 85.8 (80.7–91.0)	32/64, 50.0 (42.6–57.4)	129/177, 72.9 (66.3–79.4)

Note—PPV = positive predictive value. NPV = negative predictive value.

**Table 3:** Factors affecting diagnostic accuracy in knee stress radiographies evaluated by method 4.

	Accurate Cases (TP + FN = 129)	Error Cases (FP + TN = 48)	Odds Ratio (95% CI) *	*β* Value †	*p* Value ‡
Age—y	28.85 ± 10.68	27.29 ± 10.00	1.016 (0.983–1.054)	0.015	0.380
Male gender—no. (%)	104 (80.62)	40 (83.33)	0.832 (0.328–1.930)	−0.184	0.681
Interval between stress radiography and MRI—d	19.08 ± 13.75	24.08 ± 13.39	0.975 (0.952–0.998)	−0.025	0.035
Flexion angle on stress view—°	37.82 ± 11.36	40.24 ± 10.74	0.980 (0.951–1.010)	−0.020	0.201
Intercondylar distance on stress view—mm	4.54 ± 3.68	4.52 ± 3.46	1.000 (0.915–1.101)	0.002	0.971
Force-joint distance on stress view—mm	38.53 ± 11.48	42.84 ± 11.96	0.968 (0.938–0.996)	−0.033	0.032
Force-joint angle on stress view—°	−10.31 ± 9.28	−11.05 ± 9.50	1.009 (0.973–1.047)	0.009	0.637

Note—TP = true positive, FN = false negative, FP = false positive, TN = True negative, CI = confidence interval. *Estimates for categorical data are unadjusted odds ratios, and 95% confidence intervals are two-sided and were calculated with the use of Fisher’s exact tests. Estimates for continuous data are mean differences, and 95% confidence intervals were calculated with the use of *t*-tests. † Estimates are beta values of logistic regression models. When the factor increases by 1, the accuracy of method 4 will increase by $e^{\beta }-1$. ‡ Estimates for categorical data were calculated with the use of Fisher’s exact tests. Estimates for continuous data were calculated with the use of *t*-tests.

**Figure A1:**
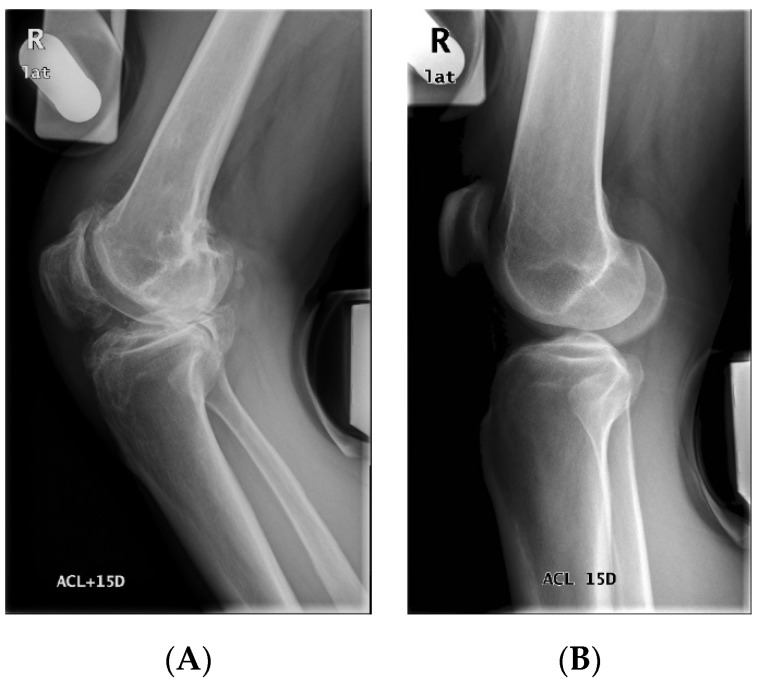
The original images of two unsuccessful cases are shown here. In these cases, the segmentation results of the femoral condyles were inappropriate due to (**A**) severe degenerative changes of the knee joint, and (**B**) the fuzzy margin of the medial femoral condyle on the radiograph.

## Data Availability

The data presented in this study are available on request from the corresponding author.
